# Pulsation of visible vessel or adherent clot in duodenal ulcer may indicate pseudoaneurysm: Case series

**DOI:** 10.1097/MD.0000000000032819

**Published:** 2023-02-03

**Authors:** Jiayu Ju, Ziyao Cheng, Qingliang Zhu, Mingming Deng, Hailong Zhang

**Affiliations:** a Department of Gastroenterology, The Affiliated Hospital of Southwest Medical University, Luzhou, Sichuan Province, China.

**Keywords:** angiography, duodenal ulcer, endoscopy, pseudoaneurysm

## Abstract

Owing to the anatomical characteristics of the duodenum, gastroduodenal artery (GDA) pseudoaneurysms can be caused by duodenal ulcers, which may rupture and lead to massive or repeated bleeding. Pseudoaneurysms are usually diagnosed using enhanced computerized tomography or angiography, whereas endoscopic signs have rarely been reported.

**Patient concerns:**

Three patients aged 18 to 83 years with bleeding duodenal ulcers and GDA pseudoaneurysms were treated.

**Diagnosis:**

All patients had symptoms of gastrointestinal bleeding, and endoscopy revealed duodenal ulcers with visible vessels or adherent clot pulsations. Angiography confirmed the presence of a GDA pseudoaneurysm, which had an adjacent relationship with the duodenum.

**Interventions:**

The GDA pseudoaneurysm was embolized in each patient.

**Outcomes:**

Through transcatheter arterial embolization, endoscopic treatment, and traditional treatment, hematemesis or melena was gradually stopped and all patients were discharged.

**Lessons:**

The pulsation of visible vessels or adherent clots observed during endoscopy in patients with duodenal ulcer may indicate the formation of a gastroduodenal artery pseudoaneurysm. Therefore, we suggest that such patients receive enhanced computerized tomography or angiography to assess whether they need timely endovascular intervention treatment to avoid bleeding caused by a pseudoaneurysm.

## 1. Introduction

The morbidity and mortality of upper gastrointestinal bleeding caused by duodenal ulcers are higher than those of gastric ulcers. Endoscopic treatment is still the first choice for patients with bleeding peptic ulcers because it can help clarify the causes of bleeding and achieve endoscopic hemostasis at the same time. According to the Forrest classification, patients with high-risk bleeding stigmata (Forrest Ia to IIb) should receive endoscopic hemostasis. However, owing to the special location of the duodenum and poor visual field after bleeding, endoscopic therapy still faces a great challenge, especially in patients with active bleeding ulcers, large ulcers (size > 1.5 cm), and ulcers with large-sized nonbleeding vessels (diameter > 2 mm), who have a high risk of rebleeding after endoscopic therapy.^[1]^ Transcatheter arterial embolization (TAE) is recommended when endoscopic hemostasis fails owing to its minimal invasiveness and effectiveness. In recent years, some studies have suggested that prophylactic transcatheter arterial embolization can reduce the risk of rebleeding in patients with a high risk of endoscopic treatment failure.^[2,3]^

Two typical signs of bleeding vessels are responsible for TAE. One is contrast agent overflow; it is not difficult to understand that this sign corresponds to an ulcer classified as Forrest Ia or Forrest Ib. Another sign is pseudoaneurysm formation. As the duodenal wall is thin and adjacent to the gastroduodenal artery (GDA), the pulsation of the pseudoaneurysm may appear as a pulsation of the visible vessel or an adherent clot in the duodenal ulcer. In this article, we report 3 consecutive cases of duodenal ulcer with bleeding, which had the pulsatile sign of visible vessels or adherent clots during endoscopy, and gastroduodenal artery pseudoaneurysms were confirmed by subsequent angiography.

## 2. Case report

### 2.1. Case 1

**Figure 1.**
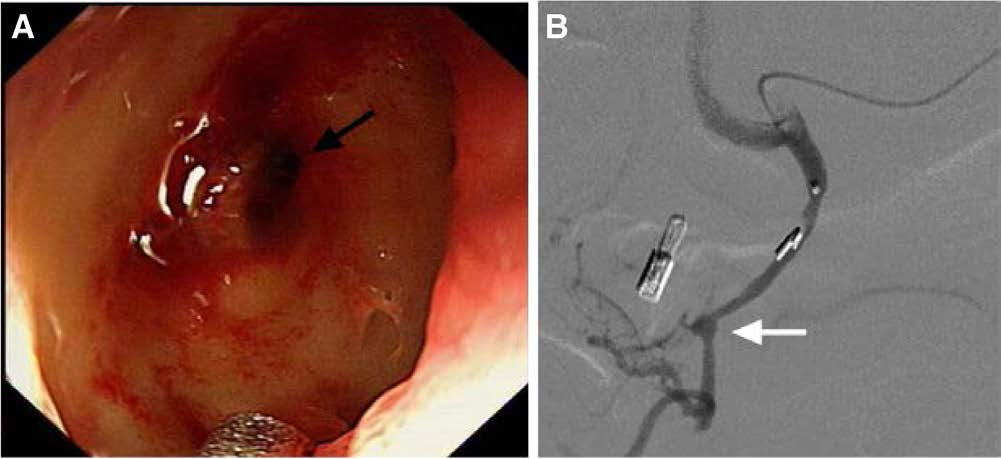
(A) Endoscopic view showed a deep concave ulcer at the junction of D1 and D2 with blood clot pulsating on its surface (black arrow); (B) Gastroduodenal angiography confirmed the gastroduodenal pseudoaneurysm (white arrow). D1 = the first part of the duodenum, D2 = the second part of the duodenum.

An 83-years-old female presented with hematemesis 4 days after eating hard food. She visited the local hospital immediately and was diagnosed with duodenal bulb ulcers by endoscopy. Although the patient was treated with hemostatic drugs, melena persisted, and dyspnea developed; therefore, she was transferred to our hospital for further treatment. She had obvious symptoms of anemia such as dizziness, fatigue, and palpitations. Her medical history included hypertension and chronic obstructive pulmonary disease. Routine laboratory examination revealed a hemoglobin level of 72 g/L, platelet count of 128 × 10^9^/L, and albumin 29.4 g/L. Upon physical examination, she was conscious, but had shortness of breath. Because her general condition was poor, the patient received conservative care, such as proton pump inhibitors and nutritional support. However, on the third day of hospitalization, the patient experienced re-bleeding with shock and underwent endoscopy immediately. Endoscopy revealed a deep ulcer in the duodenal bulb with a pulsating blood clot on its surface. Because the patient could not tolerate the discomfort caused by endoscopy under surface anesthesia, endoscopic hemostasis could not be performed. Angiography was performed owing to the high rebleeding rate. Gastroduodenal angiography revealed a pseudoaneurysm of the GDA. We used micro coils to embolize the distal and proximal trunks of the pseudoaneurysm. Angiographic control confirmed complete exclusion of the pseudoaneurysm. The patient was discharged 1 week later without hematemesis or melena (Fig. 1).

### 2.2. Case 2

**Figure 2.**
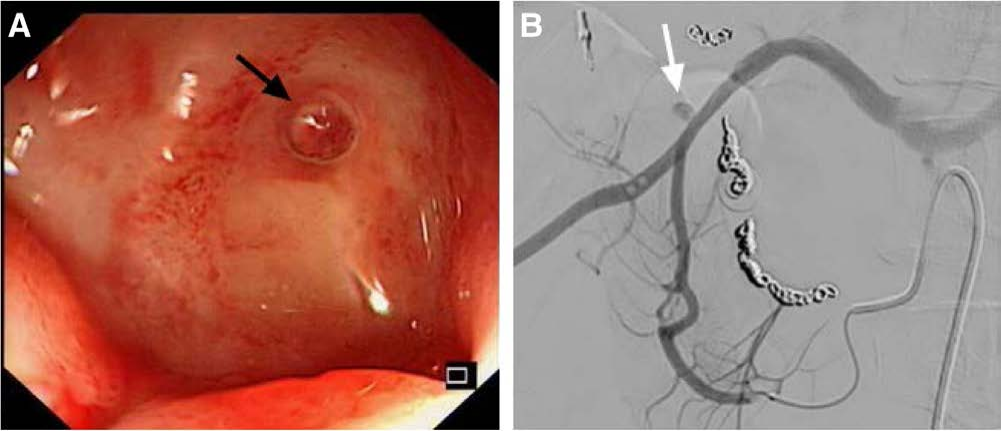
(A) Endoscopic view showed a 2 × 2cm ulcer at the junction of D1 and D2 with vessel pulsating (black arrow); (B) Mesenteric angiography confirmed the gastroduodenal pseudoaneurysm supplied by gastroduodenal artery (white arrow). D1 = the first part of the duodenum, D2 = the second part of the duodenum.

An 18-years-old male started melena after eating spicy food 5 days prior, but he did not pay attention to it. He was diagnosed with a duodenal ulcer and underwent TAE at another hospital because of aggravation of hematemesis and melena. However, melena persisted with a progressive decrease in hemoglobin level, so he was admitted to our hospital. At initial presentation, his systolic and diastolic blood pressures were 86 mm Hg and 52 mm Hg, respectively, although his heart rate did not significantly increase. His hemoglobin level was 51 g/L and physical examination revealed tenderness under the xiphoid process. Blood transfusion, proton pump inhibitors, and liquid resuscitation were administered immediately, and at the same time, emergency endoscopy was performed, revealing a 2 × 2 cm ulcer in the anterior aspect of the duodenal bulb with vessel pulsating on the surface. Hemoclipping and thermocoagulation are risky, and the ulcer was treated with diluted norepinephrine to cover the entire ulcerated area. But melena still existed and a subsequent complete blood count indicated a 25 g/L hemoglobin loss the next day, angiography was then performed and found a pseudoaneurysm in the main trunk of the GDA supplied by the superior mesenteric artery, micro coils were used to embolize the distal and proximal trunk of the pseudoaneurysm successively. The pseudoaneurysm could not be observed on angiography after embolization, indicating that the vascular intervention treatment was successful (Fig. 2).

### 2.3. Case 3

**Figure 3.**
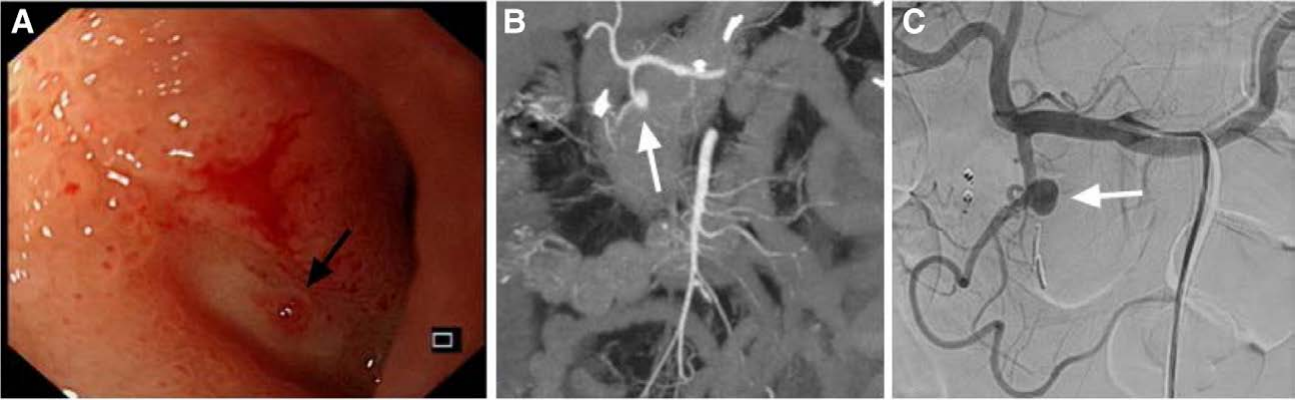
(A) Endoscopic view showed a white oval moss ulcer at the junction of D1 and D2 with vessel pulsating on its surface (black arrow); (B) Abdominal CT angiogram showed a significantly enhanced gastroduodenal pseudoaneurysm from the GDA (white arrow); (C) Gastroduodenal angiography confirmed the gastroduodenal pseudoaneurysm (white arrow). CT = computerized tomography, D1 = the first part of the duodenum, D2 = the second part of the duodenum, GDA = gastroduodenal artery.

A 65-years-old female was admitted to the outpatient department with tar stools for 5 days. She had a history of repeated abdominal pain and distension for 8 years without any systematic examination. She had hypertension, no drinking history, diabetes, or cardiovascular and cerebrovascular diseases. Endoscopy revealed a white oval moss ulcer in the front part of the duodenal bulb with a pulsating vessel on its surface in the outpatient department, and the patient was immediately admitted to the inpatient department for treatment. On admission, her vital signs were stable, with a heart rate of 90 beats/minutes, blood pressure of 105/49 mm Hg, and a hemoglobin level of 67 g/L. Physical examination revealed mild tenderness during the xiphoid process. Due to the pulsatile sign of the visible vessel on the ulcer surface under endoscopy, abdominal computerized tomography (CT) angiography was performed, which revealed a 0.8 × 1.0 cm gastroduodenal artery pseudoaneurysm near the pancreatic head area. She underwent TAE, and angiography revealed a pseudoaneurysm of the main trunk of the GDA. After embolization with micro coils, subsequent completion angiography confirmed the complete exclusion of the pseudoaneurysm (Fig. 3).

## 3. Discussion

The GDA mostly originates from the common hepatic artery, runs 1 to 2 cm from the posterior and upper part of the first part of the duodenum (D1), and travels caudally behind the anterior part of D1, which indicates an adjacent relationship between the GDA and duodenum. The junction between D1 and the second part of the duodenum (D2) is the closest point between the duodenal wall and GDA.^[4]^ Therefore, under normal conditions, we observed slight pulsation of the duodenum during endoscopy. Duodenal ulcers indicate that the mucosa has been damaged by acid and inflammation, the GDA may invade, especially in patients with repeated massive bleeding, and gastroduodenal artery pseudoaneurysms may arise. To date, all duodenal ulcers with gastroduodenal artery pseudoaneurysms have been reported to occur at the junction between D1 and D2. Sharma described the same pulsating signs^[5–7]^ which is consistent with our report. The reason is that the duodenal wall is thin and adjacent to the GDA, particularly at the junction of D1 and D2, which is the closest part, so the main trunk of the GDA is most often destroyed by duodenal ulcer, and pseudoaneurysm may occur subsequently.

A visible vessel or adherent clot represents a clot or thrombus of the pseudoaneurysm. When blood flows into the cavity of the pseudoaneurysm, pulsation can be observed. In contrast to the normal pulsation of the duodenal wall, the pulsation of a duodenal ulcer with a pseudoaneurysm is centered on visible vessels or adherent clots, whose intensity is more obvious, or just the visible vessel or adherent clot pulsates; therefore, we can distinguish it from a normal pulsation of the duodenal wall. Unfortunately, we could not provide endoscopy videos for a more intuitive understanding. However, gastroduodenal pseudoaneurysms were confirmed in 3 consecutive cases by subsequent angiography, suggesting that gastroduodenal pseudoaneurysm during endoscopy may have the special sign mentioned above. Angiography of the third case showed that the GDA pseudoaneurysm was biased to the contralateral side of the marked clip, possibly because the patient underwent angiography 7 hours later after endoscopy was completed. We speculated that the pseudoaneurysm was larger during endoscopy than during angiography. During endoscopy, the aneurysm cavity was close to the marked clip; however, due to the use of hemostatic drugs and thrombosis in the pseudoaneurysm after endoscopy, the volume of the pseudoaneurysm cavity filled with contrast agent was reduced, the shape changed, and the gastroduodenal pseudoaneurysm of the third patient was not as close to the marked clip as in the other 2 patients.

Until now, a similar sign has not been reported in gastric ulcers. The main reason for this is that the gastric wall is thicker than the duodenal wall, and the injury of the feeding artery of the stomach was less likely than that of the duodenum. A gastric artery pseudoaneurysm caused by an ulcer is usually located in a distant branch of the artery and not the main trunk. Therefore, they have a limited blood flow to the pseudoaneurysm cavity when the artery pulses and cannot cause visible endoscopic pulsation of visible vessels or adherent clots on the surface of the ulcer. However, the pseudoaneurysms in the 3 cases we reported were all located in the main trunk of the GDA; therefore, their pulsations during endoscopy were more obvious.

When gastroduodenal pseudoaneurysm is suspected, angiography is not only the gold standard for diagnosis but also a type of treatment.^[8]^ Enhanced CT is a noninvasive diagnostic modality, but its sensitivity is lower than that of angiography (67% vs 100%), and the hemostatic clip of endoscopic therapy may form an artifact that affects the diagnosis of pseudoaneurysms. multislice computed tomography and 3-dimensional (3D) magnetic resonance angiography are as effective as angiography in the diagnosis of visceral pseudoaneurysms; however, they are rarely used in the study of gastroduodenal pseudoaneurysms.^[9]^

Endoscopic hemostasis is the first-line therapy for duodenal ulcer bleeding; however, bleeding in patients with gastroduodenal artery pseudoaneurysms is difficult and risky. One reason for this is that the duodenal bulb is narrow and can be easily deformed. When the ulcer is bleeding, vision is so poor that it is difficult to operate. Another reason is that it is sometimes difficult to control bleeding of the main trunk of the GDA by hemoclipping or thermocoagulation, even with an over-the-scope clip.^[1,10,11]^ Thanks to advances in catheters and embolization materials, an increasing number of studies have concluded that angiography and embolization techniques are safe and effective and can be used as alternatives to surgery, especially for patients who cannot tolerate surgery.^[1]^ It is worth noting that there is a rare complication of embolization coil migration. Several case reports have reported that the coil migrates to the duodenal lumen after embolization of a gastroduodenal artery pseudoaneurysm, which mostly occurs at the junction of D1 and D2. A possible reason is that the coil can partially or completely migrate into the duodenal lumen through the pseudoaneurysm as the bowel moves, which indirectly confirms that the visible vessel or adherent clot is a break of the pseudoaneurysm by which the pseudoaneurysm communicates with the duodenum, and pulsation caused by the GDA pseudoaneurysm is more likely to occur at the junction of D1 and D2.^[12–16]^

## 4. Conclusion

In our opinion, the pulsation of visible vessels or adherent clots in duodenal ulcers during endoscopy in the above 3 consecutive patients eventually proved to be caused by a gastroduodenal pseudoaneurysm because of the anatomical relationship between the GDA and duodenum. Thus, the pulsation of visible vessels or adherent clots observed during endoscopy in patients with duodenal ulcers may indicate the formation of a gastroduodenal artery pseudoaneurysm. Under the above circumstances, we suggest that patients undergo enhanced CT or angiography to detect whether gastroduodenal artery pseudoaneurysm is present and receive timely endovascular intervention treatment to avoid massive or intractable bleeding caused by pseudoaneurysm. However, the number of cases observed was small, and more clinical cases are needed for further validation.

## Author contributions

**Conceptualization:** Jiayu Ju, Mingming Deng, Hailong Zhang.

**Data curation:** Jiayu Ju, Ziyao Cheng, Qingliang Zhu, Mingming Deng, Hailong Zhang.

**Formal analysis:** Jiayu Ju, Ziyao Cheng, Qingliang Zhu, Mingming Deng.

**Supervision:** Hailong Zhang.

**Writing – original draft:** Jiayu Ju, Hailong Zhang.

**Writing – review & editing:** Jiayu Ju, Qingliang Zhu, Mingming Deng, Hailong Zhang.
